# Enhancing ICU-to-Ward Handover Documentation Through a Human Factors Approach

**DOI:** 10.7759/cureus.111090

**Published:** 2026-06-18

**Authors:** Finian M O'Malley, Amelia Street, Kush Deshpande

**Affiliations:** 1 Intensive Care, St George Hospital, Sydney, AUS; 2 Critical Care, St George Hospital, Sydney, AUS; 3 School of Clinical Medicine, University of New South Wales, Sydney, AUS

**Keywords:** audit, discharge, human factors, intensive care, intensive treatment unit, quality improvement, transfer of care

## Abstract

Background

Discharge from the ICU to the ward can be associated with negative outcomes. Local guidelines mandate documenting the occurrence and time of verbal handover between the ICU team and ward team to reduce this danger. However, adherence is inconsistent and incomplete.

Objective

This study aimed to improve the frequency and completeness of documented ICU-to-ward verbal handover using a human factors-informed intervention. The outcomes assessed were documentation-related process measures rather than handover quality or patient-centered clinical outcomes.

Methods

A closed-loop quality improvement project was conducted in a tertiary ICU in Sydney, Australia. Baseline data were collected over one week for all patients discharged to the ward (n=42 ICU discharges). Discharges home and patient deaths were excluded. A multifaceted intervention was implemented over two days, comprising a visual prompt, verbal reinforcement during a department meeting, and digital dissemination. The intervention was informed by human factors principles, aiming to reduce cognitive load and improve the reliability of safety-critical tasks. Re-audit was conducted four weeks later over another one-week period (n=43). Categorical data were analyzed using chi-squared (χ²) testing with significance set at p=0.05. One key limitation is that this study assessed documentation compliance and did not examine handover quality or patient outcomes.

Results

Documentation of verbal handover improved from 25/42 (59.5%) to 37/43 (86.0%), an absolute improvement of 26.5 percentage points (95% CI: 8.4 to 44.6; p=0.006). Documentation of handover timing improved from 9/25 (36.0%) to 30/37 (81.1%), an absolute improvement of 45.1 percentage points (95% CI: 22.4 to 67.7; p<0.001).

Conclusion

A simple, low-cost intervention informed by human factors principles significantly improved compliance with ICU discharge handover standards. By introducing environmental prompts and reducing reliance on memory-dependent processes, adherence to a safety-critical documentation process improved. More research is needed to assess the impact on patient care and the sustainability of change. This approach has the potential to be scalable, sustainable, and transferable to other high-risk transitions of care.

## Introduction

Transitions of care are widely recognized as a major contributor to preventable patient harm [1]. Discharge from the ICU to a general ward represents a particularly vulnerable transition. Studies demonstrate associations between suboptimal handover and increased rates of adverse events, readmissions, and mortality. Although findings from this heterogeneous qualitative literature are not always statistically significant, the themes and links to adverse patient outcomes resulting from poor handover are consistent [1-3]. The complexity of ICU patients, combined with the shift from high-intensity monitoring and low staff-to-patient ratios to ward-based care, necessitates clear and reliable communication between teams.

Structured handover processes have been shown to improve communication quality and clinical outcomes. For instance, a systematic review by van Sluisveld et al. demonstrated that standardized handover interventions improve clarity and reduce information loss [4]. Similarly, large-scale interventions such as the I-PASS study have shown reductions in medical errors following implementation of structured handoff protocols [5].

As shown by our initial audit, adherence to handover standards in routine practice remains inconsistent. This can be understood through a human factors framework, which emphasizes the interaction between clinicians, tasks, and the systems in which they operate [6]. In high-pressure environments such as the ICU, cognitive load, interruptions, and competing priorities contribute to variability in performance. In this context, poor adherence typically reflects system pressures rather than knowledge deficits, and educational interventions alone are often insufficient to produce reliable change; approaches that redesign the working environment to support clinician performance are therefore more likely to succeed [6].

Local guidelines require that all patients discharged to the ward receive a doctor-to-doctor verbal handover, with documentation of both the occurrence and timing of this handover. This study aimed to evaluate baseline adherence to these documentation standards and to implement a human factors-informed intervention to improve the reliability of ICU discharge handover documentation. The study assessed documentation compliance as a process measure; handover quality and patient outcomes were not directly evaluated.

This study was previously presented as a poster and oral presentation at the BMJ International Forum on Quality and Safety in Healthcare in Canberra, 2025.

## Materials and methods

A closed-loop quality improvement project was conducted in a tertiary ICU in Sydney, Australia. The unit manages a mixed medical and surgical population and operates within a high-acuity, high-turnover environment. This project was conducted as a quality improvement initiative and was exempt from formal ethics review in accordance with institutional policy. This study received no external funding. The intervention was implemented using existing departmental resources at no additional cost.

Audit standards were derived from local ICU guidelines, which mandate a verbal handover between the ICU and receiving ward medical teams at the time of discharge and the documentation of this handover in the patient record and of the time of handover.

Eligible discharges were identified using a report generated by the hospital's electronic clinical information system, listing all patients discharged from the ICU during each one-week audit period. For each patient, the electronic medical record and the standardized ICU discharge proforma were reviewed for documentation of verbal handover. The discharge proforma, which was in use unchanged during both audit periods, includes a dedicated field for recording the time of handover and the clinician to whom the handover was given (e.g., the receiving ward team doctor). Data were extracted by a single investigator using identical criteria across both audit cycles; the investigator was not blinded to the audit phase.

Baseline data were collected from ICU discharge summaries over a one-week period. A total of 42 discharge summaries were analyzed.

Inclusion criteria

Patients discharged from the ICU to a hospital ward were included in the study.

Exclusion criteria

Patients were excluded from the analysis in the case of death, direct discharge home, or transfer to another healthcare facility.

Primary outcome

The primary outcome was the presence of documented verbal handover.

Secondary outcome

The secondary outcome was the presence of documented handover time.

Intervention

**Figure 1:**
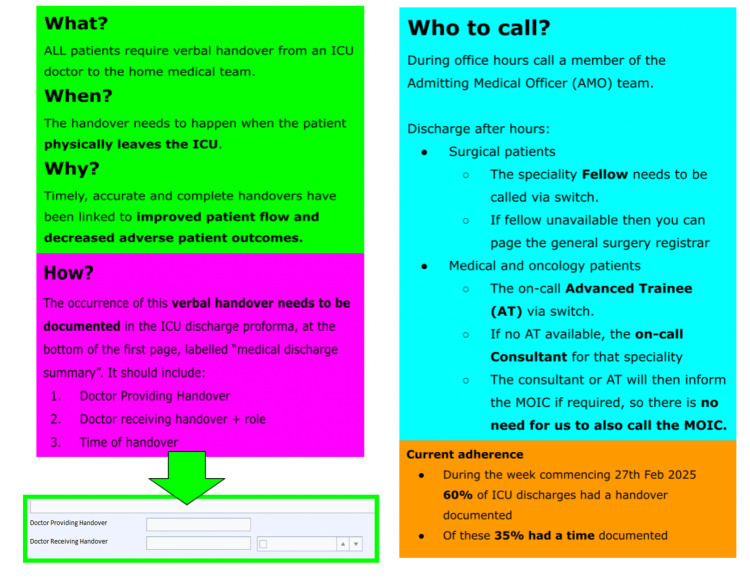
Intervention poster containing guidelines on ICU-to-ward discharges

The intervention was designed using human factors principles to reduce reliance on memory and provide point-of-care prompts. A multifaceted intervention was implemented over two days, comprising a visual prompt in the form of a poster displaying ICU handover guidelines placed in the doctors’ office (Figure 1), verbal reinforcement through discussion of the guidelines during clinical handover sessions, and digital reinforcement via distribution of the poster on a secure digital messaging platform.

Re-audit

Four weeks after the initial audit, post-intervention data were collected over one week using identical methodology (n=43). Categorical variables were analyzed using the chi-squared (χ²) test, performed using GraphPad QuickCalcs (GraphPad Software, San Diego, CA, USA). Statistical significance was set at p<0.05. Absolute differences in proportions are reported with 95% CIs.

## Results

A total of 85 ICU discharge summaries were analyzed across both audit cycles.

Primary outcome: documentation of verbal handover

**Figure 2:**
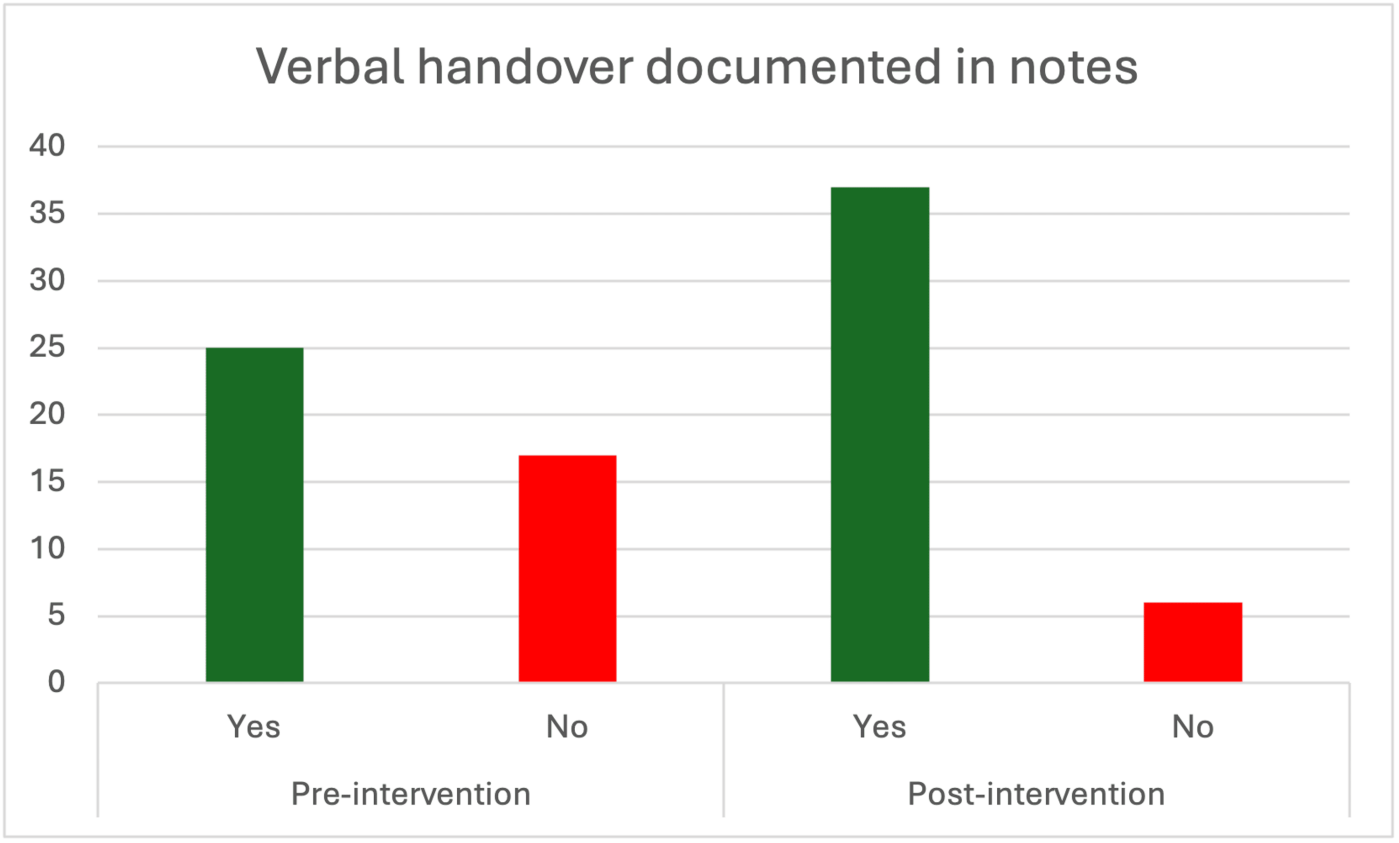
Primary outcome: documentation of verbal handover pre- and post-intervention

Pre-intervention, 25/42 (59.5%) patients had documented verbal handover. Post-intervention, this increased to 37/43 (86.0%), an absolute improvement of 26.5 percentage points (95% CI: 8.4 to 44.6; χ²=7.57, p=0.006) (Table 1) (Figure 2).

**Table 1:** Documentation in notes of verbal handover and time of handover pre- and post-intervention, including test statistic and p-value

	Pre-intervention		Post-intervention		Absolute difference, percentage points (95% CI)	Chi-square (χ²) test statistic	p-value
Documentation in notes	Yes	No	Yes	No			
Verbal handover	25	17	37	6	26.5 (8.4 to 44.6)	7.57	0.006
Time of handover	9	16	30	7	45.1 (22.4 to 67.7)	12.99	<0.001

Secondary outcome: documentation of handover timing

**Figure 3:**
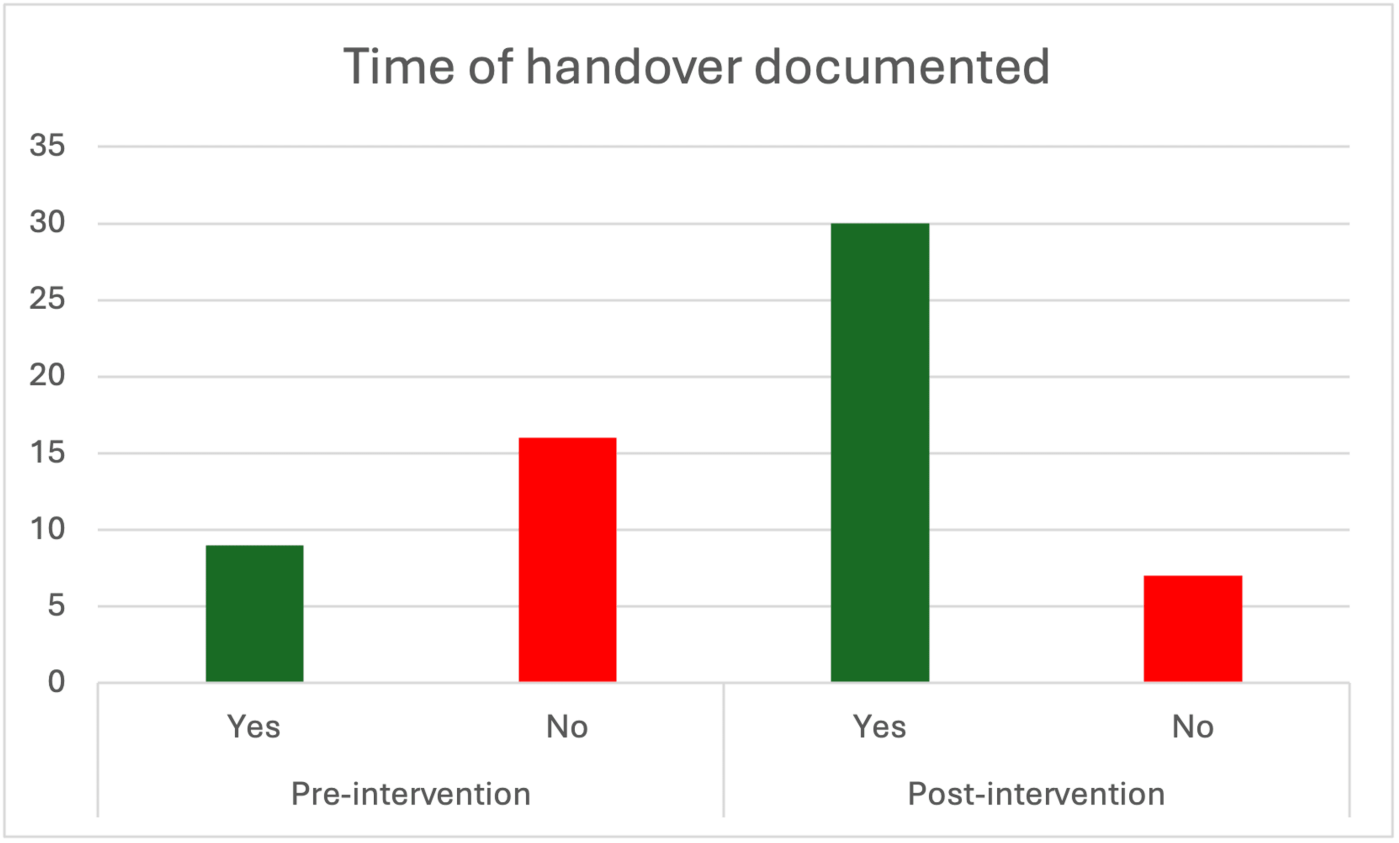
Secondary outcome: documentation of handover timing pre- and post-intervention

Among those with documented handover, documentation of timing improved from 9/25 (36.0%) to 30/37 (81.1%), an absolute improvement of 45.1 percentage points (95% CI: 22.4 to 67.7; χ²=12.99, p<0.001) (Figure 3).

Although the CIs are wide, reflecting the modest sample size, both exclude no effect and are consistent with a clinically meaningful improvement in documentation compliance.

## Discussion

This study demonstrates that a simple, low-cost intervention significantly improved compliance with ICU discharge handover documentation standards. The magnitude of improvement observed suggests that relatively small changes to the clinical environment can produce meaningful gains in safety-critical behaviors.

A key strength of this project lies in its grounding in human factors principles. Traditional approaches to improving guideline adherence often rely on education alone; however, evidence consistently shows that educational interventions in isolation produce modest and poorly sustained changes in clinical behavior [6]. Knowledge deficits are rarely the primary barrier: clinicians typically know what is required but fail to execute under conditions of high workload, interruption, and competing demands [6,7]. In such environments, memory-dependent tasks are vulnerable to omission regardless of training. A human factors approach instead modifies the system in which behavior occurs, externalizing requirements through point-of-care prompts and reducing dependence on prospective memory so that the correct action becomes easier to perform than to omit [8].

In ICU settings, clinicians operate under high cognitive load, with frequent interruptions and competing priorities [7]. Under such conditions, memory-dependent tasks may be prone to omission. The intervention addressed this by externalizing key information through visual prompts, thereby reducing reliance on working memory. This aligns with established human factors principles that emphasize designing systems to support human performance rather than expecting perfect behavior [8].

The use of environmental cues and digital prompts acted as behavioral “nudges,” increasing the salience of handover requirements at the point of care. Behavioral science literature demonstrates that such nudges can significantly influence decision-making without restricting autonomy [9].

Repetition across multiple communication channels further enhanced effectiveness. Delivering the same message visually, verbally, and digitally increases the likelihood of uptake and retention, a strategy widely used in safety-critical industries [10].

These findings are consistent with existing literature on ICU discharge processes. A systematic review by Lin et al. identified that interventions targeting ICU admission and discharge practices can improve communication and reduce variability in care [11]. Similarly, van Sluisveld et al. demonstrated that structured handover interventions improve the quality and consistency of information transfer [4].

The I-PASS study provides strong evidence that improving handover processes can reduce medical errors and adverse events [5]. While the present study focused on documentation rather than structured handover content, it addresses a critical prerequisite for safe communication, ensuring that handover occurs reliably. A potential extension of the present study could include prompting to use a structured handover tool, although adherence to this would be more difficult to assess.

Moreover, qualitative work has highlighted a persistent gap between ICU and ward teams, with discontinuity in communication contributing to patient risk [1]. Improving documentation may also enhance accountability and continuity of care, supporting both medical and nursing teams during patient transitions [12]. This study was conducted in a real-world clinical environment, enhancing its relevance and applicability. The intervention was resource-efficient and easily implementable.

However, several limitations should be considered. The study was conducted in a single center with a relatively small sample size and a short follow-up period, limiting generalizability and precluding assessment of sustained change. Most importantly, this study measured documentation compliance, a process measure, rather than the quality or content of handover communication or patient-centered outcomes. Improved documentation does not necessarily indicate that handovers were more complete, accurate, or clinically effective, and no inference can be drawn regarding adverse events, readmissions, or other patient outcomes. Data were extracted by a single unblinded investigator, and inter-rater reliability was not formally assessed; however, the binary, objective nature of the outcome measures (presence or absence of documentation in a structured proforma field) limits the potential for ascertainment bias. Additionally, a Hawthorne effect may have contributed to behavioral change [13]. Future blinded re-audit cycles, ideally incorporating measures of handover content quality and patient outcomes, would help distinguish true and durable behavioral change from observer-related effects.

Sustainability is critical for long-term impact. Measures implemented include continued display of visual prompts and incorporation of handover expectations into junior doctor induction. Embedding these processes within routine workflows reduces the risk of regression. A re-audit is planned to measure the sustainability of this change.

The intervention is highly transferable. Similar human factors-informed approaches could be applied to other high-risk transitions, including theater-to-ward handover, emergency department referrals, and inter-specialty communication. Given its low cost and minimal resource requirements, this approach is particularly relevant to resource-limited settings.

## Conclusions

A human factors-informed intervention significantly improved compliance with ICU discharge handover documentation standards. By reducing reliance on memory and introducing environmental prompts, adherence to a safety-critical documentation process was improved. This low-cost, easily implemented approach offers a practical model for improving documentation at high-risk transitions of care. However, the findings reflect short-term improvements in documentation behavior; whether these translate into improved handover quality, communication, or patient outcomes was not assessed. Repeat audit cycles are required to evaluate sustainability, the frequency with which behavioral prompts must be reinforced, and ultimately the impact on patient care.

## References

[1] Häggström M, Asplund K, Kristiansen L (2009). Struggle with a gap between intensive care units and general wards. Int J Qual Stud Health Well-being.

[2] Wibrandt I, Lippert A (2020). Improving patient safety in handover from intensive care unit to general ward: a systematic review. J Patient Saf.

[3] Ahn JW, Jang HY, Son YJ (2021). Critical care nurses' communication challenges during handovers: a systematic review and qualitative meta-synthesis. J Nurs Manag.

[4] van Sluisveld N, Hesselink G, van der Hoeven JG, Westert G, Wollersheim H, Zegers M (2015). Improving clinical handover between intensive care unit and general ward professionals at intensive care unit discharge. Intensive Care Med.

[5] Starmer AJ, Spector ND, Srivastava R (2014). Changes in medical errors after implementation of a handoff program. N Engl J Med.

[6] Carayon P, Wetterneck TB, Rivera-Rodriguez AJ, Hundt AS, Hoonakker P, Holden R, Gurses AP (2014). Human factors systems approach to healthcare quality and patient safety. Appl Ergon.

[7] Park J, Zhong X, Dong Y, Barwise A, Pickering BW (2022). Investigating the cognitive capacity constraints of an ICU care team using a systems engineering approach. BMC Anesthesiol.

[8] Reason J (2000). Human error: models and management. BMJ.

[9] Thaler RH, Sunstein CR (2008). Nudge: Improving Decisions About Health, Wealth, and Happiness.

[10] Grol R, Grimshaw J (2003). From best evidence to best practice: effective implementation of change in patients' care. Lancet.

[11] Lin FF, Chen Y, Rattray M (2024). Interventions to improve patient admission and discharge practices in adult intensive care units: a systematic review. Intensive Crit Care Nurs.

[12] Plotnikoff KM, Krewulak KD, Hernández L (2021). Patient discharge from intensive care: an updated scoping review to identify tools and practices to inform high-quality care. Crit Care.

[13] McCarney R, Warner J, Iliffe S, van Haselen R, Griffin M, Fisher P (2007). The Hawthorne effect: a randomised, controlled trial. BMC Med Res Methodol.

